# The asymmetry and temporal dynamics of incidental letter–location bindings in working memory

**DOI:** 10.1080/17470218.2014.982137

**Published:** 2014-12-06

**Authors:** Jane V. Elsley, Fabrice B. R. Parmentier

**Affiliations:** ^a^ Faculty of Science and Technology, Bournemouth University, Poole, UK; ^b^ Neuropsychology & Cognition Group, Institute of Health Sciences Research (iUNICS) and Department of Psychology, University of the Balearic Islands, Palma, Spain; ^c^ Neuropsychology & Cognition Group, Instituto de Investigación Sanitaria de Palma, Palma, Spain; ^d^ School of Psychology, University of Western Australia, Perth, Western Australia, Australia

**Keywords:** Binding, Working memory, Episodic buffer

## Abstract

Verbal–spatial bindings are integral to routine cognitive operations (e.g., reading), yet the processes supporting them in working memory are little understood. Campo and colleagues [Campo, P., Poch, C., Parmentier, F. B. R., Moratti, S., Elsley, J. V., Castellanos, N., … Maestú, F. (2010). Oscillatory activity in prefrontal and posterior regions during implicit letter-location binding. *Neuroimage*, *49*, 2807–2815] recently reported data suggesting obligatory letter–location binding when participants were directed to remember the letters in a display (of letters in locations), but no evidence for binding when instructed to remember the filled locations. The present study contrasted two explanations for this *binding asymmetry*. First, it may result from an obligatory dependence on “where” during the representation of “what” information, while “where” information may be held independently of its contents (the *strong asymmetry* hypothesis). Second, it may constitute a snapshot of a dynamic feature inhibition process that had partially completed by test: the *asymmetrical inhibition* hypothesis. Using Campo and colleagues’ task with a variable retention interval between display and test, we presented four consonants in distinct locations and contrasted performance between “remember letters” and “remember locations” instructions. Our data supported the *strong asymmetry* hypothesis through demonstrating binding in the verbal task, but not in the spatial task. Critically, when present, verbal–spatial bindings were remarkably stable, enduring for at least 15 seconds.

Our experience of the world is based upon fully formed objects appearing within coherent scenes, yet this experience is built from collections of features (e.g., colours, shapes, and locations) that are initially processed in distinct cortical regions (Tootell, Dale, Sereno, & Malach, 1996). The cognitive system is faced with the challenge of putting this disparately processed information back together and, in the case of working memory (WM), making the resulting *bound* representations available once stimuli are no longer in view. While much experimentation has been directed toward the investigation of feature binding in visual WM, relatively little is understood about cross-domain associations between *verbal* and *spatial* representations, in spite of their prevalence during routine cognitive operations.

Verbal–spatial bindings represent an interesting theoretical case, as according to the working memory model (Baddeley & Hitch, 1974) verbal and spatial features are processed within distinct stores (the phonological loop & the visuospatial sketchpad, respectively). However, while the verbal–spatial distinction has garnered empirical support from various research traditions (e.g., behavioural selective interference effects: Logie, 1995; developmental studies: Hitch, 1990; neurological impairments: Della Sala & Logie, 1993), recent investigations suggest that their processing may not be as independent as strict modularity prescribes. Specifically, behavioural (Elsley & Parmentier, 2009) and imaging (Prabhakaran, Narayanan, Zhao, & Gabrieli, 2000) studies support the contention that verbal and spatial representations are integrated in WM. Most striking is the recent demonstration of verbal–spatial *binding asymmetry* (Campo et al., 2010) whereby when presented with arrays of consonants in distinct locations, participants bound letters and locations when directed to remember the letters, but not when directed to remember the filled locations. This asymmetrical relationship may stem from the ubiquitous assertion that spatial location holds a “special status” in visual cognition, providing a reference frame for multiple stored items, while the pattern of filled spatial locations may be held independently of their contents (Treisman & Zhang, 2006). Under this interpretation, the binding of “where” to “what” may be unavoidable when “what” information is the subject of the task (see also Jiang, Olson, & Chun, 2000; Olson & Marshuetz, 2005), while the “where” information may be stored as a pattern of unfilled locations, independently of their contents. We refer to this position as the *strong asymmetry* hypothesis.

Alternatively, the asymmetry may arise because feature bindings are temporally dynamic, changing over time with task demands or in the service of conserving mental resources (the *asymmetrical inhibition* hypothesis). Indeed, Logie, Brockmole, and Jaswal (2011) recently provided evidence for a temporally dynamic binding process whereby potentially disruptive task-irrelevant features were inhibited from consolidated bound representations. Critically, the inhibition process took longer for spatial (“where”) features (circa 1500 ms) than for visual (“what”) features (circa 1000 ms), leading the authors to suggest that: “the process of deleting or inhibiting an irrelevant and disruptive feature from VSTM and the forgetting of details from VSTM is common to location, shape, and colour but occurs at different rates” (Logie et al., 2011, p. 35). Accordingly, as a fixed retention interval (1200 ms) was assessed in Campo et al.’s (2010) study, the binding asymmetry may constitute a still-frame of a dynamic process that had only partially completed at test: Task-irrelevant letter features may have already been inhibited from location representations by 1200 ms post stimulus offset in the spatial task, while residual bindings between letters and their locations may have been present in the verbal task as the inhibition process had not yet completed. The present study aimed to distinguish between the *strong asymmetry* hypothesis and the *asymmetrical inhibition* hypothesis.

In our version of Campo et al.’s (2010) task, participants were presented with arrays of four consonants (simultaneously) in distinct locations. At test, participants judged whether a single probe letter in location represented a letter they had seen in the memory display (the *verbal task*) or a location that was occupied in the memory display (the *spatial task*). In both tasks, we compared performance across two critical probe conditions, both of which required a “yes” response. *Intact* probes consisted of a letter in its original location (binding repeated) while *recombined* probes consisted of a letter in a location originally occupied by a different to-be-remembered letter (binding switched). If verbal and spatial features are bound in WM, we should observe a performance advantage in the intact condition relative to the recombined condition, a potential difference we refer to as a *binding effect.* The theoretical interpretation of this difference was highlighted by Prabhakaran et al. (2000). Describing the difference they observed between intact (referred to as “congruent” in their study) and recombined (referred to as “incongruent” in their study) probes, they suggest that “subjects maintained the target displays in the bound condition in an integrated fashion, such that they could quickly compare the congruent probes to the similar, integrated contents of working memory. They were slower, however, when they had to reorganize the information in working memory to compare to the incongruent probes” (Prabhakaran et al., 2000, p. 87). In other words, the reason for the facilitating effect of intact relative to recombined probes reflects the idea that a comparison between a stored representation and a probe stimulus is more efficient when the two match and less efficient when the comparison involves decomposing bound representations into constituent features (see also Ecker, Mayberry, & Zimmer, 2013; Maybery et al. (2009) for a similar theoretical interpretation of this comparison). In contrast, if verbal and spatial information is processed in parallel, there should be no performance difference between intact and recombined conditions, which (aside from their bindings) are equivalent at the feature level: Both contain a letter feature and location feature that were present in the memory array (see Method section for remaining probe conditions).

In order to contrast our hypotheses regarding binding asymmetry, we assessed performance over four retention intervals (the time elapsed between display and test) as follows: 200 ms, 500 ms, 5000 ms, 15,000 ms. The *strong asymmetry hypothesis* predicts binding between letters and their spatial positions in the verbal task but no binding in the spatial task—a consequence of spatial representations being held independently of their contents. The *asymmetrical inhibition* hypothesis makes similar predictions about performance in the verbal task; however, it predicts that there may be evidence for binding between letters and locations in the spatial task at shorter but not at longer retention intervals, on the basis that task-irrelevant letter information may be inhibited from spatial representations comparatively quickly.

## EXPERIMENTAL STUDY

### Method

#### Participants

Twenty-four undergraduates from the University of Plymouth participated in the 2-hour study (2 × 1-hour sessions, 24 hours apart) for course credit (age: *M* = 32.81 years, *SD* = 10.58, 15 females). All participants reported normal/corrected vision.

#### Materials

Stimuli were presented on a 19″ computer monitor, and responses were collected via computer keyboard. The tasks were purpose written using E-Prime software (Schneider, Eschmann, & Zuccolotto, 2002). The verbal stimuli comprised eight consonants (Arial font; 48 pt), selected to differ in appearance between upper- and lower-case forms (D; F; H; J; N; Q; R; T). The spatial stimuli comprised a set of eight spatial locations placed equidistantly in a circular arrangement around a central fixation cross.

#### Design and procedure

The study took a 2 (memory task: verbal, spatial) × 4 (retention interval: 200 ms, 500 ms, 5000 ms, 15,000 ms) × 5 (probe type: intact, recombined, new-letter, new-location, new-both, described below) within-subjects design, with task order (verbal task/spatial task) blocked and counterbalanced across participants. Stimulus presentation was identical in both tasks, only the instructions differed (i.e., to remember the letters, or to remember the locations, depending on the task undertaken).

**Figure 1:**
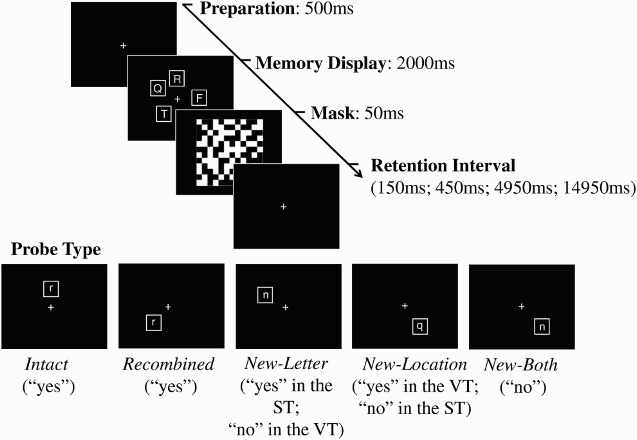
Schematic illustration of trial types in the verbal task (VT) and the spatial task (ST), with corresponding correct responses.

Each trial began with the presentation of a fixation cross (500 ms), followed by memory display (2000 ms) consisting of four white consonants (each presented in a 30 × 30-mm white frame against a black background) selected at random (without replacement) from the set of eight, each appearing in a different location randomly selected from the possible set of eight. A 50-ms visual mask then replaced the array and was followed by a blank retention interval featuring only a fixation cross for 150 ms, 450 ms, 4950 ms, or 14,950 ms (selection randomized across the tasks). Finally, participants were presented with a single probe item, which remained on screen until response (see Figure 1). Participants pressed the “j” button to indicate a yes response and the “f” button to indicate a no response (mapping reversed for half the participants). The instructions and probe conditions differed for each task, as described below.

##### The verbal task

In the verbal task, participants were instructed to memorize the letters in the display and to judge whether the single probe item represented a letter that they had seen on that trial, regardless of its location. There were three probe types requiring a “yes” response (contained a letter that had been seen on that trial): *Intact probes* consisted of a letter in its original spatial location (i.e., bindings repeated); *recombined* probes consisted of a letter that was seen on that trial that had switch locations with a different array letter (bindings switched); and *new-location probes* consisted of a letter that was seen on that trial, occupying a location that had not been filled. Probes requiring a “no” response were: *new-letter* probes, consisting of a letter that had not been presented on that trial, occupying a location that was filled by a different letter; and *both-new* probes consisting of a new letter and a new location. Array letters were presented in upper-case form while probe letters were presented in lower-case form to ensure verbal rather than visual processing. The task consisted of 240 trials, split into five blocks of 48 trials with brief rest periods administered between each block. Within each block, there were eight of each of the positive probe types in addition to 12 of each of the negative probe types (order randomized across the task). The four retention intervals (200 ms, 500 ms, 5000 ms, 15,000 ms; including the visual mask) were distributed equally across trial types and were mixed randomly within each block. Participants were instructed to be as accurate as possible while not taking too long to respond.

##### The spatial task

In the spatial task, participants were instructed to memorize the locations presented in the display and to judge whether the single probe represented a location that had been filled on that trial, regardless of its contents. Conditions requiring a “yes” response were *intact* probes, *recombined* probes, and *new-letter* probes, while those requiring a “no” response were *new-location* probes and *both-new* probes. Trial quantities were as in the verbal task.

### Results

#### *Analyses*

The Results section focuses on error rates for intact and recombined probes (as a function of attended feature and retention interval) as binding was the focus of this study*.* For completeness, analyses of all three positive probe conditions and the two negative probe conditions can be found in the Supplemental Material. Error data but not response times are reported, as the latter did not represent a sensitive measure (yielding no differences between probe conditions).

#### *Errors*

**Figure 2:**
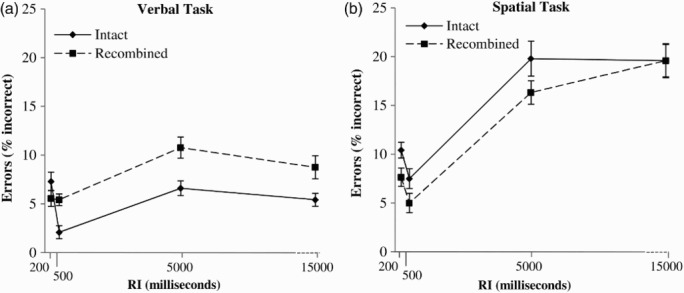
Error data (% incorrect) for intact and recombined probes as a function of retention interval in the verbal task (Panel A) and spatial task (Panel B). RI = retention interval. Error bars represent one standard error of the mean.

A 2 (memory task: verbal task; spatial task) × 4 (retention interval: 200 ms, 500 ms, 5000 ms, 15,000 ms) × 2 (binding: intact, recombined probe) analysis of variance (ANOVA) for repeated measures conducted on error data (% incorrect) indicated a significant main effect of memory task [*F*(1, 23) = 18.30, *MSE* = 238.63, *p* < .001, η^2^_p_ = .44], with fewer errors in the verbal task (*M* = 6.48, *SE* = 1.06) than in the spatial task (*M* = 13.23, *SE* = 1.63), a significant main effect of retention interval [*F*(3, 69) = 17.27, *MSE* = 97.36, *p* < .001, η^2^_p_ = .43], characterized by linear [*F*(1, 23) = 28.58, *MSE* = 106.57, *p* < .001, η^2^_p_ = .56] and cubic [*F*(1, 23) = 19.56, *MSE* = 93.28, *p* <.001, η^2^_p_ = .46] trends, and no significant binding effect [*F*(1, 23) = 0.002, *MSE* = 81.82, *p* = .96]. There was no interaction between retention interval and binding [*F*(3, 69) = 0.84, *MSE* = 77.87, *p* = .48] and no three-way interaction between factors [*F*(3, 69) = 0.59, *MSE* = 84.83, *p* = .63]; however, the interactions between memory task and retention interval [*F*(3, 69) = 7.59, *MSE* = 79.29, *p* < .001, η^2^_p_ = .25] and memory task and binding [*F*(1, 23) = 5.42, *MSE* = 88.08, *p* = .03, η^2^_p_ = .19] were both significant. The data are illustrated in Figure 2.

Further tests decomposing the interaction between memory task and retention interval indicated a significant main effect of retention interval in the spatial task [*F*(3, 69) = 16.59, *MSE* = 125.40, *p* < .001, η^2^_p_ = .42], characterized by linear [*F*(1, 23) = 27.94, *MSE* = 162.35, *p* < .001, η^2^_p_ = .55] and cubic [*F*(1, 23) = 16.77, *MSE* = 88.46, *p* < .001, η^2^_p_ = .42] trends, and a significant main effect of retention interval in the verbal task [*F*(3, 69) = 3.95, *MSE* = 51.25, *p* = .01, η^2^_p_ = .15], characterized only by a significant cubic trend [*F*(1, 23) = 8.22, *MSE* = 58.32, *p* = .01, η^2^_p_ = .26]. In sum, the error data suggested that performance was superior in the verbal task relative to the spatial task, a difference driven by steeper memory decline in the spatial task than in the verbal task. Additionally, the cubic trends present in both tasks appear to reflect performance improvements between the two shortest retention intervals consistent with other observations of an initial consolidation period for visually presented arrays lasting for approximately 200–500 ms post stimulus offset (Jiang, 2004).

Further tests decomposing the interaction between attended feature and binding indicated no evidence for binding (intact/recombined probe difference) in the spatial task [*F*(1, 23) = 1.58, *MSE* = 145.60, *p* = .22], but a significant binding effect in the verbal task [*F*(1, 23) = 10.22, *MSE* = 24.31, *p* < .001, η^2^_p_ = .31], replicating Campo et al.’s (2010) binding asymmetry finding.

### Discussion

Following a recent demonstration of verbal–spatial binding *asymmetry* (the binding of letters to locations when letters are memorized but not when locations are memorized: Campo et al., 2010); in addition to the observation that visuospatial bindings may change over time (Logie et al., 2011) this paper investigated the (a)symmetry and temporal dynamics of verbal–spatial bindings in WM.

We reasoned that Campo et al.’s (2010) binding asymmetry effect may represent a *true* asymmetry where the pattern of stored locations are held independently of their contents when sufficient for completing the task (Treisman & Zhang, 2006), while memory for letter identity is (perhaps only initially: Logie et al., 2011) dependent on the representation of spatial locations. We contrasted this *strong asymmetry hypothesis* with an alternative whereby the asymmetry may instead represent a static snapshot of a dynamic binding process through which task-irrelevant features are inhibited from stored representations—a process that reportedly may take longer to accomplish for “where” information than for “what” information (Logie et al., 2011), and one that may have only been partially completed by the time memory was probed in Campo et al.’s (2010) task (the *asymmetrical inhibition* hypothesis). We reasoned that, on the basis that “what” information may be inhibited from stored representations relatively quickly, letters may be bound to task-relevant spatial representations during the initial phases of WM maintenance (i.e., at shorter retention intervals than that used by Campo et al., 2010), but that these bound representations may diminish relatively quickly following an inhibition process. In contrast, “where” information may take comparatively longer to remove from “what” representations such that given more time between display and test, task-irrelevant spatial information may be deleted from verbal representations too.

Using a single-probe change detection paradigm (Campo et al., 2010) with a variable retention interval, we found that, regardless of retention interval duration, letters were bound to their spatial locations when participants were instructed to remember the identity of the letters (the verbal task), but not when participants were instructed to remember the spatial locations that letters were presented in (the spatial task), providing support for the *strong asymmetry* hypothesis. We suggest that, consistent with Treisman and Zhang's (2006) assertion, it may not be possible to store letters without their spatial positions, while the pattern of filled locations may be maintained independently of their contents when this suffices for task completion (see also Jiang et al., 2000). An alternative account of the data may be to suppose that the observed asymmetry is recall driven. In line with Campo et al.’s (2010) task, consonants were always presented within frames across both our verbal and spatial tasks. Consequently, spatial locations formed an inbuilt feature of verbal representations (as letters always appeared in distinct locations), while potentially, the verbal and spatial features may have been decoupled in the spatial task, with recall operating on the basis of frames (devoid of their contents). Under this interpretation, perhaps binding occurred in the spatial task too, but was not evident at the time of test by virtue of the selective recall of the frames (or the inhibition of the irrelevant verbal feature at the point of decision). While our data cannot rule out this possibility, we would argue that it is unlikely to account for the asymmetry observed here as according to existing research, the inhibition of an irrelevant feature is a process that takes time to manifest (Logie et al., 2011; Treisman & Zhang, 2006). Our data clearly indicate an absence of binding in the spatial task, even at the shortest epoch.

Interestingly, our data appear to suggest that once established, verbal–spatial bindings survived in memory for 15 s, a remarkable finding given that participants were free to engage in the rote rehearsal of a consonant “list” if they had chosen to do so. The fact that binding (and consequently the storage of twice as many features) was apparent in the task that yielded fewest errors (the verbal task) may seem counterintuitive. However, it is consistent with earlier demonstrations suggesting that spatial information may be encoded automatically with “what” features and, as a consequence, come along with no penalty to available resources (see Finke, Bublak, Neugebauer, & Zihl, 2005). In summary, it would appear that spatial information is not so readily inhibited from verbal representations.

One potential explanation for the longevity of verbal–spatial bindings is the natural interdependence between verbal and spatial associations in routine cognitive operations like reading. Orthographic coding theories of word recognition (e.g., the interactive-activation model, McClelland & Rumelhart, 1981; the MROM model, Grainger & Jacobs, 1996) explain the need to map abstract letter identities to their positions within words via “slot”-based accounts (to avoid reading the word “top” as “pot”). Thus spatial information may form an integral, ingrained part of verbal processing with long-lived associations as a consequence. A second possible explanation for the longevity of binding in our study relative to others may relate to its binary nature. Binary bindings may be more stable—that is, be maintained over longer intervals—than three-way bindings such as those measured by Logie et al. (2011) and Treisman and Zhang (2006). As noted by Hommel (2004), “feature conjunctions commonly seem to be represented by several separate, binary bindings, a loose network of clusters rather than one master file” (p. 495). Consequently, reverting to a binary association by inhibiting an irrelevant “third feature” may represent a return to “default” and be accomplished with greater efficiency than inhibiting an element of a binary association.

As pointed out by Ecker et al. (2013), there are two broad alternatives regarding the origin of binding asymmetries, which can be summarized as follows. First, some features are always primary within a given modality following a set hierarchy. These features are salient/dominant and are not bound to others by virtue of this. Second, rather than having a hierarchy of features, it is the salience of one feature relative to another in a particular task that is key—in other words, asymmetry is down to the relative difficulty of processing each feature (bootstrapping a hard feature to an easier feature to boost the memorability of the harder feature). Some have argued for the latter (Maybery, Leung, Terne, van Valkenburg & Parmentier, 2012; Melara & Mounts, 1993; Sobel & Cave, 2002; Theeuwes, 1994; van den Berg, Cornelissen & Roerdink, 2008). There is, however, evidence questioning the role of feature difficulty/discriminability. For example, Maybery et al. (2009) reported asymmetric binding for auditory verbal and spatial features (binding when location was attended, but not when sound identity was the attended feature) even though feature discriminability/difficulty was controlled for. Additionally, Campo et al. (2010) observed the same asymmetrical pattern of performance as that in our study even though, in their experiment, participants performed (at least numerically) better in the spatial task than in the verbal task. We have also found evidence of an asymmetry in the binding of shapes and locations (binding when participants attend the “what” but not when they attend the “where”) in a task in which accuracy was greater for the spatial task (Elsley & Parmentier, 2014). In sum, the data from the present study would seem to support the notion that task difficulty is not the only defining factor of binding asymmetry (see also Ecker et al., 2013, for a discussion of this point).^1^

^1^To further assess the impact of feature difficulty on binding (a)symmetry, an analysis of our data at the 500-ms lag (where error rates were roughly equivalent in the verbal and spatial tasks) was conducted. A 2 (memory task: verbal task; spatial task) × 2 (binding: intact; recombined probe) ANOVA for repeated measures confirmed the absence of a main effect of memory task [*F*(1, 23) = 1.53, *MSE* = 97.83, *p* = .23], no main effect of binding [*F*(1, 23) = 0.10, *MSE* = 43.30, *p* = .76], but a significant interaction between factors [*F*(1, 23) = 4.29, *MSE* = 47.65, *p* = .05, η^2^_p_ = .16], derived from a significant binding effect in the verbal task [*F*(1, 23) = 4.00, *MSE* = 33.33, *p* = .05, η^2^_p_ = .15] that was absent in the spatial task [*F*(1, 23) = 1.30, *MSE* = 57.61, *p* = .27]. Thus, consistent with Campo et al.'s (2010) original finding, when feature difficulty was equated, the binding asymmetry remained.


More broadly, one challenge for theoretical explanations of feature binding is to explain how, across tasks where stimulus presentation was identical, and only the instructions differed, binding occurred when the “what” features were to be remembered, but not when the “where” features were the subject of the task. Precisely how and when features are held in integrated format remains to be established, but the emerging picture is that the binding of “where” to “what” may be unavoidable and relatively long-lived, at least when verbal–spatial binary associations are assessed. Whether other types of incidental binary association are as long-lived would speak to the issue of whether verbal–spatial bindings hold a special status in WM processing and would form an interesting extension to this work.

### Supplemental material

Supplemental (content) is available via the “Supplemental” tab on the article's online page (http://dx.doi.org/10.1080/13506285.2014.982137).
